# A dyadic perspective on disease coping experiences and support needs in chronic heart failure patients and informal caregivers: a qualitative meta-synthesis

**DOI:** 10.3389/fcvm.2026.1780966

**Published:** 2026-03-27

**Authors:** Lei He, Yingnan Zhao, Xiangyu Wang, Xiaoqing Shi, Chun Li

**Affiliations:** 1 Department of Cardiology, The First Affiliated Hospital of Soochow University, Suzhou, China; 2 Department of Nursing, The First Affiliated Hospital of Soochow University, Suzhou, China; 3 School of Nursing, Medical College of Soochow University, Suzhou, China

**Keywords:** chronic heart failure, dyads, informal caregiver, meta-synthesis, qualitative research

## Abstract

**Objectives:**

To systematically evaluate the experiences of chronic heart failure patients and their informal caregivers in jointly managing the disease, clarify their support needs, and provide evidence for developing targeted interventions.

**Design:**

A qualitative meta-synthesis.

**Methods:**

We systematically searched PubMed, Embase, CINAHL, Web of Science, Cochrane Library, CNKI, Wanfang, and VIP for qualitative studies examining the dyadic coping experiences of patients with chronic heart failure and their caregivers. The quality of the literature was evaluated according to the JBI Critical Appraisal Checklist for Qualitative Research, and the included literature was integrated and analyzed using the pooled integration approach.

**Results:**

A total of 16 studies were included. 55 findings were aggregated into 13 categories and further synthesized into 4 overarching themes: divergent perspectives on illness and care; psychosocial resource depletion; disruptions in family intimacy; and the need for multidimensional support to reconstruct personal meaning.

**Conclusions:**

Healthcare professionals should pay close attention to differences in thinking, the degree of depletion of psychological resources, the tension in family intimacy, and supportive needs during the dyadic coping process between patients with chronic heart failure and their caregivers. Based on this, individualized interventions can be developed, leveraging digital technologies and platforms to enrich intervention formats and content, thereby enhancing collaborative disease management between both parties.

**Systematic Review Registration:**

https://www.crd.york.ac.uk/PROSPERO/view/CRD420251243342, CRD 420251243342.

## 1 Introduction

Chronic heart failure (CHF) represents the severe manifestation and advanced stage of various cardiovascular diseases, characterized by a prolonged course and recurrent acute exacerbations, imposing a substantial global disease burden. Statistics indicate that CHF affects over 65 million people worldwide (1). In the United States, approximately 6.7 million adults suffer from heart failure, with projections reaching 8.7 million by 2030 (2). In China, annual new cases of heart failure reach 3 million, with total hospitalization costs amounting to 22.98 billion CNY, gradually becoming one of the primary consumers of healthcare resources (3, 4).

The lengthy and complex treatment process for CHF poses significant challenges for patient self-care. Informal caregivers (individuals such as family members or friends who provide unpaid care without professional training) play a critical role in daily assistance, health management, emotional support, and information coordination, effectively alleviating the care burden on the healthcare system (5). As a result, most patients rely on informal caregivers for daily support after discharge. However, the severe symptom burden and self-care demands place both CHF patients and their informal caregivers in a chronic high-stress environment, subjecting them to substantial caregiving burdens and psychological strain. This not only compromises the quality of care but also has downstream effects on patient health and the caregiver-patient relationship (6).

Consequently, research increasingly views CHF patients and their informal caregivers as a relational unit, examining how they jointly navigate challenges affecting the unit's overall health (7). A recent study exploring dual self-care in heart failure synthesized published quantitative and qualitative research on the topic. It found that shared perspectives between patients and informal caregivers exert a protective effect, and that pre-illness relationships influence disease experience and management (8). However, this review primarily synthesizes existing literature without delving specifically into qualitative studies, failing to fully capture the myriad challenges and supportive needs faced by this interdependent team during daily caregiving tasks.

To address this gap, we conducted a qualitative meta-synthesis of relevant literature. This approach allowed us to explore the authentic experiences and needs of CHF patients and their informal caregivers as they cope with the disease together. These findings lay the groundwork for developing targeted, dyad-focused interventions in future research.

## 2 Methods

### 2.1 Design

The Enhancing Transparency in Reporting the Synthesis of Qualitative Research guidelines and the PRISMA guidelines guided the reporting of this meta-synthesis. The protocol has been registered in the International Prospective Register of Systematic Reviews (PROSPERO) (identification number: CRD 420251243342).

### 2.2 Data sources and searches

First, appropriate search terms were identified based on the purpose of the study as well as its content: “Heart Failure/Cardiac Failure/Heart Decompensation/Myocardial Failure”, “Caregivers/parents/spouses/family members/couples/children/dyadic coping”, “Qualitative research/grounded theory/ethnography/phenomenological research/narrative research”. The literature search was conducted in November 2025 and included articles published from the inception of the database to 30 November 2025. We then systematically searched 8 electronic databases, including five English-language databases: PubMed, Embase, Cochrane Library, Web of Science, Cumulative Index to Nursing Allied Health Documentation (CINAHL), and three Chinese databases: CNKI, Wanfang, VIP. Grey literature was not included, as this meta-synthesis focused on published peer-reviewed qualitative studies to ensure transparency, reproducibility, and quality standards. The sample search strategy of PubMed is shown in Table 1.

**Table 1:** Search strategy in pubMed.

Search number	Search terms
#1	“Heart Failure” [Mesh] OR “Cardiac Failure” [Title/Abstract] OR “Heart Decompensation” [Title/Abstract] OR “Myocardial Failure” [Title/Abstract]
#2	“Caregivers” [Title/Abstract] OR “parents” [Title/Abstract] OR “spouses” [Title/Abstract] OR “family members” [Title/Abstract] OR “couples” [Title/Abstract] OR “children” [Title/Abstract] OR “dyadic coping” [Title/Abstract]
#3	“Qualitative research” [Title/Abstract] OR “grounded theory” [Title/Abstract] OR “ethnography” [Title/Abstract] OR “phenomenological research” [Title/Abstract] OR “narrative research” [Title/Abstract]
#4	#1 AND #2 AND #3

### 2.3 Eligibility criteria

Inclusion Criteria: Population (P): Patients and their caregivers aged ≥18 years with CHF; Interest of phenomena (I): This review considered studies that examine experiences and feelings among CHF patients and their caregivers during disease management; Context (C): In nursing homes, communities, hospitals, or patients’ homes; Study design (S): Included qualitative research and mixed methods research from which a qualitative component could be extracted. Included research using any qualitative methodology, including but not limited to phenomenological research, descriptive qualitative research, grounded theory, ethnography, etc.

Exclusion criteria: Conference proceedings, master's and doctoral theses, and internal publications; Duplicate publications; Non-Chinese or non-English literature where full text is unavailable, or data is incomplete; Literature rated as Grade C in quality assessment.

### 2.4 Data selection and extraction

Two reviewers independently conducted the literature search, screened, and extracted data. Search results were imported into EndNote 21 for management and analysis. After duplicate checking, titles and abstracts were read carefully against the inclusion and exclusion criteria. Then, the full-text was screened to confirm eligible articles. The key extracted contents included: first author name, publication year, country, study population, research methodology, phenomena of interest, and major findings. In instances where discrepancies arose, discussions with the third reviewer were employed to reach a consensus.

### 2.5 Methodological appraisal

The Joanna Briggs Institute (JBI) Evidence-Based Healthcare Centre's Quality Assessment Criteria for Qualitative Research was used to assess the quality of included studies (9). Study quality was independently assessed by two researchers using a 10-item tool, with each item rated as “Yes,” “No,” “Unclear,” or “Not Applicable.” The overall quality of each study was then graded as A (fully compliant), B (partially compliant), or C (fully non-compliant). Any disagreements were resolved through discussion or adjudication by a third researcher. Only studies rated Grade A or B were included in the final analysis.

### 2.6 Method of synthesis

Two researchers trained in qualitative research methodology, grounded in the philosophical underpinnings and methodologies of various qualitative approaches, first extracted, translated, and interpreted the research findings from the included literature. They then analyzed the themes within the literature and the original statements therein, proceeding to code, compare, and synthesize similar findings into new categories. Finally, based on the logical relationships among these categories, they further consolidated them into higher-level, more concise themes. When disagreements arose between the two researchers, they consulted with experts experienced in qualitative research and members of the research team for arbitration. No qualitative software was used; all coding and synthesis were performed manually.

## 3 Results

### 3.1 Search results

**Figure 1:**
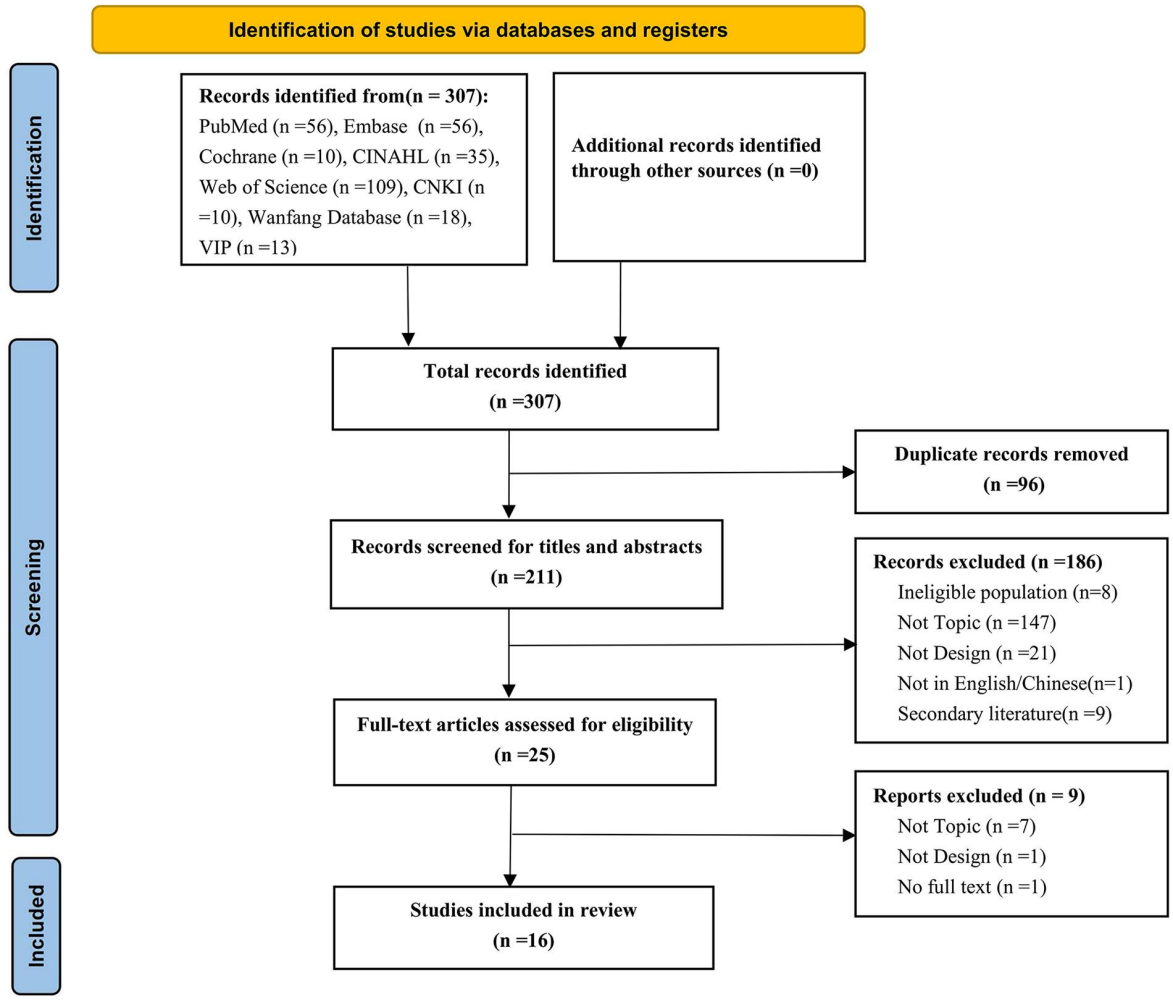
PRISMA flowchart showing the study selection process.

A preliminary search of domestic and international databases yielded 307 articles, including 266 in English and 41 in Chinese. After removing duplicates, 211 studies were screened based on title and abstract. The remaining 25 full texts were assessed according to the eligibility criteria, resulting in 16 included studies (Figure 1).

### 3.2 Characteristics of included studies

A total of 16 articles were published between 2012 and 2024, of which 14 studies were conducted in Western countries and 2 in mainland China (Table 2). Most were phenomenological research (*n* = 9), followed by mixed-method studies (*n* = 3), grounded theory (*n* = 2), ethnographic research, and descriptive qualitative research (*n* = 1 each). A total of 223 dyads were included. There were 541 participants in total.

**Table 2:** Basic characteristics of the included literature (*n* = 16).

Author/year	Country	Design	Study population		Phenomena of interest	Major findings	
			Patient (N)	Caregiver (N)		Themes (N)	Details
Egerod et al. (24)	Denmark	Grounded Theory	7	7	Caregiver experiences and coping strategies during left ventricular assist device implantation in CHF patients	2	Supporting the patient Suffering and self-preservation in close relatives
Retrum et al. (14)	USA	Phenomenological Research	17	17	Congruence and incongruence between CHF patients and their caregivers	4	Managing illness Perceived care needs Perspectives about the future of HF End-of-life issues
Liljeroos et al. (23)	Sweden	Phenomenological Research	19	19	Perceived care needs in CHF patients and their caregivers	2	Dyads perceive a need for continuous guidance through the different phases of the illness trajectory Dyads perceive a need to share burden and support with each other and others
Kitko et al. (12)	USA	Phenomenological Research	47	47	Discrepancies between CHF patients and caregivers	3	Disease management Health care issues End-of-Life decisions
Buck et al. (13)	USA	Mixed-method research	27	27	CHF patients and caregivers engage in participation in disease management	3	According to established patterns across the life course of the relationship According to whether it's day to day care or symptom management By mobilizing the help of a third party as consultant
Im et al. (10)	Canada	Phenomenological Research	12	7	Understanding disease, uncertain experiences, and perspectives on End-of-Life discussions in advanced disease among CHF patients and caregivers	3	Understanding of illness Illness understanding and experiences of uncertainty Influence of uncertainty on end-of-life communication
Neo et al. (16)	Singapore	Grounded Theory	30	11	Caregivers nursing experiences for CHF patients with left ventricular assist devices	3	Permanent and recurrent losses (Long-Term Challenges) Mutuality and connectedness (Long-Term Needs) Need for holistic care
Kim et al. (18)	USA	Mixed-method research	17	17	Cognitive and emotional experiences of CHF patients and their caregivers in coping with the disease	3	Health beliefs of dyads were characterized by acceptance and optimism, but also pessimism Negative emotions influenced the dyadic experience The closeness of their interpersonal relationships influenced their contributions to managing HF
Tulloch et al. (20)	Canada	Phenomenological Research	16	16	Perceived needs and expectations of cardiovascular disease patients and their spouses	8	Emotional and communication disconnect Overprotection of patient Role changes Adjustment to lifestyle changes Positive relationship changes Practical resources Sharing with peers Relationship enhancement
Risbud et al. (21)	USA	Phenomenological Research	17	17	Positive behaviors, cognitive and social factors in shared health management by CHF patients and their families	3	Management of HF was perceived as successful when individuals in a dyad both received support from a shared care network when strength of the interpersonal relationship and love were the main motivators for care, dyads reported a positive outlook on quality of life with HF The family caregivers’ own health conditions affected the dyadic relationship and perceived success with HF management
Golan et al. (11)	Israel	Phenomenological Research	7	7	Strategies for Couples Regarding Left Ventricular Assist Device Implantation	5	Burden versus balanced dependence Shared versus conflicting illness narrative Fear recognized and discussed versus Dyadic versus individual humor Adapted versus avoided intimacy
Xu et al. (15)	China	Phenomenological Research	21	21	Experiences of Chinese Family Caregivers in Symptom Management and Seeking Medical Care During HF	3	Responsible symptom managers: leading the home-based symptom management Powerless anchors: care-seeking is a torturous journey Carrying the weight forward: responsibility combining with emotional burnout,
Younas et al. (19)	Italy/Spain/USA	Mixed-method research	50	50	Experiences of Social Support Among Caregivers of CHF Patients	3	Familial network for tangible assistance and moral support Social sources for emotional refuge Factors hindering personal sense of perceived social support
Bjornsdotti et al. (25)	Iceland	Ethnographic Research	10	10	How CHF Patients Manage Their Condition with Caregiver Support	3	Practices of attunement in relations Becoming among difficulties Off track-difficult to attune around self-care
Graven et al. (22)	USA	Phenomenological Research	11	11	Self-Care Challenges Experienced by Rural CHF Patients and Caregivers and How They Address These Issues	4	HF self-care components, namely, maintenance, symptom monitoring, and management Environment Caregiver contributors Dyadic contributors
Li et al. (17)	China	Descriptive Qualitative Research	10	10	Home Care Needs for Patients and Primary Caregivers Following Left Ventricular Assist Device Implantation	3	Self-management needs Sports rehabilitation needs Social support needs

CHF, chronic heart failure; HF, heart failure;.

### 3.3 Results of methodological appraisal

The quality of the included studies was generally good (Table 3), with adequate quotes supporting the researchers’ interpretations, which were also described in detail.

**Table 3:** Methodological quality evaluation of the included literature (*n* = 16).

Author/year	Q1	Q2	Q3	Q4	Q5	Q6	Q7	Q8	Q9	Q10	Total
Egerod et al. (24)	CT	Y	Y	Y	Y	N	N	Y	Y	Y	B
Retrum et al. (14)	CT	Y	Y	Y	Y	N	N	Y	Y	Y	B
Liljeroos et al. (23)	Y	Y	Y	Y	Y	Y	N	Y	Y	Y	B
Kitko et al. (12)	CT	Y	Y	Y	Y	N	Y	Y	Y	Y	B
Buck et al. (13)	CT	Y	Y	Y	Y	N	N	Y	Y	Y	B
Im et al. (10)	Y	Y	Y	Y	Y	N	Y	Y	Y	Y	B
Neo et al. (16)	CT	Y	Y	Y	Y	Y	Y	Y	Y	Y	A
Kim et al. (18)	CT	Y	Y	Y	Y	N	N	Y	Y	Y	B
Tulloch et al. (20)	Y	Y	Y	Y	Y	Y	Y	Y	Y	Y	A
Risbud et al. (21)	CT	Y	Y	Y	Y	N	N	Y	Y	Y	B
Golan et al. (11)	Y	Y	Y	Y	Y	Y	N	Y	Y	Y	B
Xu et al. (15)	CT	Y	Y	Y	Y	N	N	Y	Y	Y	B
Younas et al. (19)	CT	Y	Y	Y	Y	Y	N	Y	Y	Y	B
Bjornsdotti et al. (25)	Y	Y	Y	Y	Y	Y	Y	Y	Y	Y	A
Graven et al. (22)	Y	Y	Y	Y	Y	N	N	Y	Y	Y	B
Li et al. (17)	CT	Y	Y	Y	Y	N	N	Y	Y	Y	B

Q1: Whether the philosophical foundation aligns with the methodology? Q2: Whether the methodology aligns with the research question or objectives? Q3: Whether the methodology aligns with the data collection methods? Q4: Whether the methodology aligns with the representativeness of the data and the data analysis methods? Q5: Whether the methodology aligns with the interpretation of results? Q6: Whether the researcher's own background is explained from the perspectives of cultural context and values? Q7: Whether the researcher's influence on the study or the study's influence on the researcher is articulated? Q8: Whether the research subjects are typical and adequately represent the population and its perspectives? Q9: Whether the research complies with current ethical standards? Q10: Whether conclusions are derived from the analysis and interpretation of data?

Y, yes; N, no; CT, can't tell.

### 3.4 Synthesis results

After repeated reading, analysis, comparison, and refinement of the literature, 55 research findings were identified. Similar findings were grouped into 13 categories and ultimately consolidated into 4 outcomes. The integrated results are shown in Table 4.

**Table 4:** Meta-synthesis results.

Themes	Subthemes
Differences in perception and philosophy	Symptom perception
	Adherence
	Views on life and death
Psychosocial resource depletion	Fear and anxiety
	Stigma and isolation
Imbalance in family relationships	Role maladjustment
	Lack of constructive communication
	Reduced sexual activity and impaired fertility
Support needs and meaning reconstruction	Practical information resources
	Improving healthcare
	Family and peer support
	Workplace
	Spiritual beliefs

#### 3.4.1 Theme 1: differences exist in multiple views

##### 3.4.1.1 Sub-theme 1: symptom perception

The irregular progression of the disease and its atypical clinical manifestations lead to differences in symptom perception between the two (patient: “I don't have shortness of breath, it's just the flu.” caregiver: “It seemed like flu, but she lacked… it was always accompanied by shortness of breath. I thought it was her heart”) (10) [Denmark]. However, the main reason lies in differences in knowledge structures, resulting in a failure to promptly capture the body's warning signals (caregiver: “Last time, he was gasping for breath, and his eyes rolled back. But this time it was edema, so I didn't pay much attention”) (11) [Israel].

##### 3.4.1.2 Sub-theme 2: adherence

They often disagree on whether to strictly adhere to the treatment plan (caregiver: “The doctor said with this new treatment, he should be able to walk.” patient: “She nags me all day to walk more”) (12), particularly regarding medication adherence (caregiver: “She always forgets to take her medicine.” patient: “If I want to take my meds, I'll take them”) (13). Patients feel caregivers constantly judge them based on their own mental templates (patient: “I drink a bit more water, and she gives me grief, but I didn't drink a whole bottle; I wouldn't let myself get dehydrated”) (12) and fundamentally fail to understand them (patient: “She doesn't get that I just don't have the stamina”) (12).

##### 3.4.1.3 Sub-theme 3: views on life and death

The constraints of views on life and death and family bonds lead some families to emphasize the overall significance of a dignified passing, reluctant to discuss death extensively (family member: “He's not at that stage yet. From my experience, his physical condition is still quite good”) (10), and even prohibit medical staff from mentioning it in the patient's presence (family member: “I told the doctor not to use the term “hospice care’ around him.”) (14). Conversely, patients themselves often attach little importance to the length of life (patient: “Is it because we're breathing that we prolong life?”) (14), reject medical technology to sustain life (patient: “We don't want to live on life support from machines”) (10), and accept death with equanimity, unwilling to burden their children (patient: “I'm prepared for death and strive to organize my life accordingly”) (10).

#### 3.4.2 Theme 2: psychosocial resource depletion

##### 3.4.2.1 Sub-theme 1: fear and anxiety

CHF is a progressive disease characterized by periodic deterioration and unpredictable disease trajectories. Once related clinical manifestations appear, caregivers are plunged into anxiety and panic (caregiver: “I live in constant fear. When he gets up at night, I immediately get up to see what's wrong with him”) (15), maintaining constant vigilance over the patient (caregiver: “Every hour, every day, I have to think about his illness”) (16). This anxiety intensifies during episodes of worsening symptoms or deterioration (caregiver: “When she said her heart was pounding, I was truly terrified”) (16). Even though the left ventricular assist device offers patients a “second chance at life” with significant health benefits, both patients and caregivers remain fearful of device failure (patient: “It saved my life, but it's still just a machine. I never know when it might break down or explode”) (16). They worry about postoperative complications (caregiver: “Is the bleeding from my husband's abdominal wound going to get infected?”) (17) and even feel anxious about the external cables and batteries restricting daily life, directly affecting sleep [caregiver: “I can't sleep unless he replaces the battery” (11); patient: “When sleeping, I usually only dare to lie on my right side and never sleep deeply, afraid of crushing the cables or accidentally dropping the device on the floor”] (17). Over time, the body remains in a state of heightened alertness, making individuals prone to emotional exhaustion. This exhaustion can lead them to downplay or even ignore signals from their own bodies (patient: “This isn't a life-or-death issue, it's not like cardiac arrest”) (10), attributing such concerns to caregivers taking things out of context or exaggerating the facts (patient: “I'm telling you she's exaggerating. She has anxiety”) (11).

##### 3.4.2.2 Sub-theme 2: stigma and isolation

The chronic physical toll of illness and the uncertainty surrounding its unknown symptoms have robbed patients of their most basic daily functional abilities (patient: “Every day I trouble my wife to get up early and dress me”) (16), rendering them unable to work (patient: “I can't work—I work for three days, then end up hospitalized for a week”) (16). Compounded by the public's insufficient understanding of the disease (patient: “When I take public transportation in the morning… When I walk, people bump into me constantly, and everyone stares at me”) (16), leading to feelings of stigmatization and self-doubt (patient: “I can't care for them, I can't hug my grandchildren”) (16). This gradually fosters extreme thoughts (patient: “Don't worry, I'll just die”) (18). Some caregivers refuse to accompany patients in public due to severe physical symptoms or visible medical devices, also experiencing stigma (caregiver: “This is a left ventricular assist device, so wherever you are, whenever the alarm wants to go off, it will—on the bus, in a taxi, even at the mall”) (16). The prolonged demands of caregiving and fear of disease recurrence disrupted caregivers’ established social structures, undermining their internal stability and external sense of security (caregiver: “You have to take care of them, then work for them. You have to do things”) (16). They are forced to withdraw from social circles (caregiver: “Because I have to care for him, I've had to reduce contact with others. I have no life of my own”) (11), triggering isolation (caregiver: “I feel disconnected from society”) (17). Single-parent families experience deeper abandonment and loneliness (caregiver: “I'm an only child. If something happens to her, I'll be alone.” patient: “She doesn't care about me, so I'll be alone. What can I do?”) (19) [Italy/Spain/USA].

#### 3.4.3 Theme 3: imbalance in family relationships

##### 3.4.3.1 Sub-theme 1: role maladjustment

Individuals often juggle multiple social roles, and the sudden onset of illness disrupts the existing balance between patients and caregivers, hindering role transitions (patient: “Now it's your turn to work and earn money”) (16). Male patients, in particular, feel they have transformed from the family's primary breadwinner into its main burden, experiencing guilt (patient: “It's my heart problem that's now become her problem; I feel so sorry”) (20) and a sense of disrupting the caregiver's normal life (patient: “She should have a normal life, but now I'm an obstacle to her”) (20). Yet faced with the uncertainty of the disease's course and the fear and pain of potentially losing a loved one, patients are forced into the role of the vulnerable, often becoming overly protected (patient: “I feel like a puppet on strings”) (20). As the illness worsens, the time consumed by actual caregiving increases, while personal free time diminishes [caregiver: “I'm basically his nurse” (21); caregiver: “I'm just a slave”] (11), forcing them to abandon former pleasures (caregiver: “I'm the driver, the eyes, the doctor. I've had to give up all the joy and fun in my life”) (21), leading to severe physical and mental exhaustion (caregiver: “Last year I also developed a terrible heart condition”) (19). Their own need to be cared for is neglected (caregiver: “I've become irritable, tense, and stressed, but I still have to care for others. What am I supposed to do?”) (20).

##### 3.4.3.2 Sub-theme 2: lack of constructive communication

Maintaining good communication between the two parties helps them jointly cope with the stress caused by illness and alleviate negative emotions. Conversely, negative communication exacerbates psychological burdens, amplifies conflicts, and widens the emotional distance between them. When patients express their feelings and concerns to caregivers but fail to receive corresponding emotional support, both parties enter a state of ineffective communication (patient: “Are you angry with me?” caregiver: “Did you see that truck over there?”) (20). Communication conflicts frequently arise between some patients and caregivers over whether immediate hospital care is necessary [caregiver: “I tell her to go to the hospital sooner, and she flies into a rage at me” (15); caregiver: “He forbids calling a doctor, but if you have to call an ambulance to take him to the ER, he'll fight it tooth and nail”] (13). They may resort to avoidance (patient: “I never want to discuss anything about me with her again”) (15), or even develop escape thoughts (patient: “Enough, I'm leaving this place”) (20). Some patients avoid discussing their condition to spare caregivers (patient: “Her job and other responsibilities keep her busy enough”) (14), while caregivers’ delayed understanding of disease progression and severity stems from patients’ communication avoidance (caregiver: “If she told us what she feared or worried about, things wouldn't worsen, but she just won't speak up”) (20). These persistent communication barriers, combined with the progressive limitations of disease, frequently result in significant frustration (patient: “it's limited my activities… things I'd like to do… I can't do. I get real frustrated…. caregiver: sometimes we'll plan to go somewhere and then I can't go. It's so frustrating… the last time I was in the hospital, he really felt bad, because there was nothing he could do… he gets frustrated…. I know sometimes I get frustrated”) (22). The primary reasons for some individuals’ poor role adjustment capabilities are twofold: on one hand, they care for the patient out of a sense of duty (caregiver: “Because I'm the eldest, I felt it was my job…”) (18), yet their thoughts and actions remain inconsistent; on the other hand, they measure the experience by its value, perceiving caregiving as offering no tangible rewards (caregiver: “It's a burden, and you don't get what you need”) (11).

##### 3.4.3.3 Sub-theme 3: reduced sexual activity and impaired fertility

Sexual activity can trigger sympathetic nervous system arousal and increase cardiac load. Considering health concerns, couples often proactively avoid intercourse (patient: “I have no strength; to keep me alive, we need to avoid sex”) (11) [Israel] or cease it entirely (patient: “Sexual activity… has completely stopped for two years. Completely stopped”) (16). Rooted in traditional cultural influences and inheritance mechanisms, young patients not only face sexual challenges but also endure the pressure of permanent infertility (patient: “I feel useless as a woman who can't conceive normally”) (16) [Singapore]. Even when spouses attempt to understand the pain of fertility difficulties, they cannot conceal their frustration (spouse: “How to put it… pregnancy is practically impossible. First, she's on medication. Second, she has a device implanted inside her, so…”) (16).

#### 3.4.4 Theme 4: support needs and meaning reconstruction

##### 3.4.4.1 Sub-theme 1: practical information resources

As the illness worsens, both CHF patients and caregivers require practical and professional information resources, which are essential for dyadic self-care (patient/caregiver: “I need more information”) (23) and address the new problems that arise (patient: “If I get worse, don't you think it would be helpful to have more information about what's happening and what to do next?”) (14), particularly regarding emergency procedures [caregiver: “Lucy had a seizure at home. I'd never seen anything like it before and panicked” (24); caregiver: “The equipment suddenly stopped working and the patient collapsed. What should we do?”] (17) and physical rehabilitation (caregiver: “No one told us how to exercise at home. They just said to take it slow and not overexert ourselves”) (17). Thus, both parties desire specialized, practical information from healthcare providers rather than mere expressions of concern (caregiver: “They say, “Take care of yourself,’ but no one says, “If you can do a, b, c, that's a useful way to take care of yourself..’ Like stress-reduction techniques. A new method to help you learn how to do things, or even a support group on how to help yourself while your spouse is recovering”) (20), while also expecting changes in how information is delivered (patient/caregiver: “We need face-to-face meetings”) (20).

##### 3.4.4.2 Sub-theme 2: improving healthcare

Both expressed a desire for healthcare institutions to refine outpatient classifications (patient/caregiver: “I want a heart failure clinic”) (23) and ensure smooth communication channels (patient/caregiver: “It would be best to have the phone number for the heart failure outpatient clinic or ward”) (14). Additionally, medical institutions should conduct regular assessments of patients’ conditions and develop personalized treatment plans (caregiver: “For young people who've had heart transplants, they get up and can even recover. But for the elderly, other diseases and issues arise”) (16). Particular emphasis should be placed on addressing caregivers’ needs (caregiver: “No one pays attention to my thoughts”) (5). As primary stakeholders in the patient's health (caregiver: “This affects both of us”) (11), caregivers must be integrated into the entire medical treatment process (caregiver: “Include us in training”) (20). Furthermore, heart failure service teams should be diverse (caregiver: “It could be doctors, nurses, physiotherapists, social workers, etc.”) (23), with particular emphasis on nurse-led care (patient/caregiver: “They were very experienced and answered most questions”) (23). Efforts should be made to cultivate family nurses who provide health guidance (caregiver: “When his condition worsened, the family nurse visited him weekly to offer support”) (25).

##### 3.4.4.3 Sub-theme 3: family and peer support

Support from other family members can help patients and caregivers navigate decision conflicts to obtain optimal medical care (caregiver: “My daughter gave me incredible help. She helped me make crucial decisions to manage her father's many conditions”) (5). Peer conversations and confiding in others of the same gender facilitate emotional release and deepen mutual closeness (caregiver: “I call my mom three times a day. Together with my sister, we can spend three hours on the phone every day, for an entire year”) (5). Additionally, family members are expected to provide tangible assistance (patient/caregiver: “My husband renovated the house to accommodate the “heart companion,” and my sister helped move equipment”) (23). Beyond familial support, peer support is particularly vital for patients (patient: “Regular group meetings with others in similar situations”) (14), enabling access to more information for self-help (patient: “I found meeting people with the same issues very helpful”) (20). Simultaneously, caregivers expressed a desire for peer support to enhance their caregiving capabilities (caregiver: “I wish I knew what I could do to help him instead of pushing him away or reacting negatively”) (20).

##### 3.4.4.4 Sub-theme 4: workplace

After developing an illness, patients not only face the constant threat of unemployment (patient/caregiver: “He almost lost his job because of his left ventricular assist device”) (10), but also endure skepticism from employers (patient: “Do you have…, Can you…?”) (14). For patients, such negative external attitudes limit their participation in life situations, diminishing their sense of self-identity (patient: “I've never not worked, and I feel lost… I feel utterly useless”) (22). For caregivers, the heavy burden of care disrupts their original life trajectories, forcing varying degrees of disconnection from society (caregiver: “Since my husband fell ill and was hospitalized, I haven't worked in a long time”) (17). Over time, the direct consequence is financial strain (caregiver: “My son doesn't earn much from his part-time job. We borrowed over 50,000 yuan for his treatment, and I also have a daughter in middle school”) (17) [China]. The indirect outcome is reduced social participation for both patients and caregivers, hindering personal development.

##### 3.4.4.5 Sub-theme 5: spiritual beliefs

Religious faith serves as a pathway for both to find solace and positive experiences (patient/caregiver: “We are Catholics, very active. We study the Bible at home and find great joy in it”) (21). They believe religion helps them escape their predicament and accept harsh realities [caregiver: “We just follow God's plan. If he's lucky, he might survive…” (15); patient/caregiver: “We must… it depends on our God”] (16).

## 4 Discussion

This study employed an integrative approach to synthesize 16 literature sources examining the shared experiences, perspectives, and needs of CHF patients and their informal caregivers in coping with the disease. The analysis identified four primary themes: (1) differences in Perception and Philosophy; (2) psychosocial Resource Depletion; (3) imbalance in Family Relationships; (4) support Needs and Meaning Reconstruction.

The high uncertainty in CHF prognosis, frequent readmissions, and limited treatment options in advanced stages (26) lead to widespread psychological distress among patients and caregivers, including fear and feelings of helplessness, often falling below the threshold. Research indicates (27) that significant psychological distress persists and remains strongly correlated for both parties even one year or more after discharge. Accordingly, healthcare providers are advised to cultivate positive qualities such as gratitude and post-traumatic growth in both individuals to delay cardiac function decline and adaptive deterioration. Second, regular psychological assessments by mental health professionals are recommended. Targeted interventions based on these assessments may include self-disclosure group therapy to reduce the harm caused by long-term self-suppression and enhance intimacy between patients and caregivers. Online delivery protects privacy, minimizes exposure risks, and efficiently addresses issues (28, 29), aiming to strengthen positive beliefs, reinforce the will to survive, and improve self-regulation abilities. Additionally, when cardiac function permits, moderate outdoor exercise is recommended to experience nature's healing power. For patients with reduced exercise tolerance, virtual reality devices can be used to access nature's therapeutic effects from home, alleviating negative emotions. Finally, healthcare institutions should implement remote self-monitoring systems to provide real-time guidance for disease self-management. Caregivers should be trained in professional skills and care knowledge, utilizing remote monitoring systems to observe patients while maintaining spatial freedom for both parties (30).

This study found that CHF patients and caregivers exhibit conflicting perceptions regarding symptom perception, adherence, and views on life and death. These discrepancies primarily stem from the complexity of disease symptoms, disparities in health literacy, and unequal knowledge frameworks between both parties (31). Regarding views on life and death, caregivers often avoid in-depth discussions about death and may even discourage healthcare providers from mentioning end-of-life care (10, 14), while patients tend to face death with greater composure (10, 14). Chinese studies (15, 17) further highlight the dimension of collective family decision-making, where the protective role of family members is more pronounced. This suggests that healthcare institutions should strengthen synchronized health education, optimize communication methods, and regularly assess knowledge retention. Concurrently, education on dignified dying and end-of-life care should be integrated into health management. Healthcare providers need to consider family cultural backgrounds, appropriately guide patients and caregivers toward open communication, and involve them in treatment decisions to alleviate anxiety.

Throughout the disease coping cycle, cognitive discrepancies lead to imbalances in intimate relationships, manifesting as role maladjustment, communication barriers, and sexual dysfunction. Research indicates (32) that stable intimate relationships serve as vital coping resources. Therefore, enhancing both parties’ role adaptation and transition capabilities facilitates shared goal-setting and confidence-building, thereby strengthening intimate bonds. Healthcare providers should deliver precise, standardized, and personalized medical information tailored to family cultural backgrounds. Furthermore, CHF patients and caregivers often lack constructive communication during coping processes. From the medical perspective, factors such as timing, communication methods, language barriers, or information overload during inpatient health education can interrupt information flow, preventing effective mutual understanding and leading to disagreements. From the patient and caregiver perspective, conflicts may be closely related to gender, time of diagnosis, duration of care, educational level, and psychological distress (33). Healthcare providers are advised to facilitate communication between patients and caregivers by enhancing communication awareness, optimizing communication techniques and content, establishing clear communication channels, and reducing internal conflicts.

Sexual health remains an often-overlooked dimension of quality of life. Sexual dysfunction rates among cardiovascular disease patients reach 62.6%, yet 51.5% of healthcare providers have never offered related guidance (34, 35), primarily due to knowledge gaps and prioritization biases in treatment planning. Recommendations include: strengthening sexual health training for healthcare providers and clarifying consultation responsibilities (e.g., cardiologists, psychologists), and incorporating sexual function and fertility into routine assessments. However, due to privacy concerns and traditional cultural taboos in China, patients and their spouses often find it difficult to proactively discuss sexual issues. Therefore, sexual health counseling should be integrated into routine clinical care starting from the diagnostic phase, with personalized guidance provided based on factors such as age and treatment stage. Additionally, establishing a professional online platform to deliver standardized health education and Q&A services is recommended.

This study emphasizes that healthcare institutions need to provide personalized, practical information support to assist patients and caregivers in disease management, particularly during the transition and home care phases. Research indicates (36, 37) that effectively embedding the goal-framing effect into the development of health education manuals, production of health education videos, and writing of health news and articles significantly enhances health literacy. This approach also facilitates healthcare providers in conveying reliable, objective, and effective disease-related information while mitigating potential risks. Healthcare professionals can integrate the goal-framing effect with digital technology to develop tools that regularly assess disease severity, cardiac function classification, educational background, and information preferences. Based on these assessments, targeted information can be delivered to meet the evolving information needs of patients and caregivers throughout disease stages, thereby preventing information gaps that arise from traditional word-of-mouth communication.

This study also found that both patients and caregivers expressed a need for third-party support systems, including family members, peer support, the healthcare system, and religious support (38, 39). However, the availability and forms of third-party support vary across cultures. Western cultures often emphasize peer support and formal services (20, 23), whereas nearly 79% of CHF patients in China are elderly (3), with caregivers predominantly being aged spouses. The phenomenon of elderly care is widespread. Influenced by traditional ethical values, mutual support among elderly couples is perceived as a moral obligation inherent to the marital commitment. This contrasts with Western cultures where caregivers can access formal service support, suggesting a heavier burden of elderly care in China. Recommendations include: First, establishing community day care centers to provide respite care and alleviate caregiving burdens. Second, implementing family-based welfare subsidy systems to offer economic security for elderly care.

## 5 Limitations

The majority of included studies were rated as Grade B quality, suggesting potential bias in the integrated findings. Second, only two of the 16 included studies were conducted in China, with the remainder from Western countries. This cultural imbalance limits the transferability of findings to non-Western settings, particularly regarding culturally sensitive issues such as end-of-life communication, sexual health, and family caregiving roles—domains where our Discussion identified notable differences between Western and Chinese contexts. Furthermore, qualitative research inherently involves subjective interpretations, potentially introducing bias during data analysis and affecting the accuracy of results. Future studies are advised to adopt mixed-methods designs combining quantitative and qualitative approaches to minimize bias. Additionally, as shown in Table 3, several included studies did not explicitly report researcher reflexivity or acknowledge how the researchers’ backgrounds and assumptions may have influenced data collection and interpretation. This limited reporting of reflexivity may affect the dependability of the primary studies and, consequently, the interpretations drawn in this synthesis. Finally, by restricting inclusion to English and Chinese language publications, we may have excluded relevant studies published in other languages, which could introduce language bias.

## 6 Conclusion

The study employs a meta-synthesis approach to organize and analyze the experiences and needs of CHF patients and their caregivers during the dyadic coping process of the disease. It suggests that healthcare providers should not only ensure consistency in disease cognition between both parties but also pay attention to the degree of psychological resource depletion. Furthermore, they should maintain a balanced development of the intimate relationship between them. By strengthening individual coping skills, reducing relational discord, and expanding external support resources, the multidimensional needs of both parties can be addressed, ultimately improving their ability to cope with the disease. Future research should prioritize developing culturally adapted interventions that address communication barriers, role adaptation challenges, and resource limitations, with special consideration for rural and underserved populations.

## Data Availability

The original contributions presented in the study are included in the article/Supplementary Material, further inquiries can be directed to the corresponding authors.
